# QSAR study of some 5-methyl/trifluoromethoxy- 1*H*-indole-2,3-dione-3-thiosemicarbazone derivatives as anti-tubercular agents

**Published:** 2009

**Authors:** M. Shahlaei, A. Fassihi, A. Nezami

**Affiliations:** 1 *Department of Medicinal Chemistry, Faculty of Pharmacy, Kermanshah University of Medical Sciences, Kermanshah, I.R.Iran*; 2 *Department of Medicinal Chemistry, School of Pharmacy and Pharmaceutical Sciences, Isfahan University of Medical Sciences, Isfahan;Isfahan, I.R.Iran*; 3 *Isfahan Pharmaceutical Sciences Research Center, School of Pharmacy and Pharmaceutical Sciences, Isfahan University of Medical Sciences, Isfahan, I.R.Iran*; 4 Department of Pediatrics, Faculty of Medicine, Lorestan University of Medical Sciences, Khoramabad, I.R.Iran

**Keywords:** QSAR, Thiosemicarbazone derivaives, Principal component analysis, Anti-tuberculosis activity

## Abstract

In the present study, quantitative relationships between molecular structure and anti-tubercular activity of some 5-methyl/trifluoromethoxy-1*H*-indole-2,3-dione-3-thiosemicarbazone derivatives were discovered. The detailed application of an efficient linear method and principal component regression (PCR) for the evaluation of quantitative structure activity relationships of the studied compounds is demonstrated. Components produced by principal component analysis were used as the input for a linear model development. Results indicate a linear relationship between the principal components obtained from molecular descriptors and the inhibitory activity of this set of molecules. The maximum variance in the activity of the molecules in PCR method was 73%. The performance of the developed model was tested by several validation methods.

## INTRODUCTION

Tuberculosis (TB) is an infectious condition with high degree of mortality all over the world. According to the World Health Organization one-third of the world’s population was infected with *Mycobacterium tuberculosis* in 2006 (1). Other statistics indicate that 8 million of new cases are estimated to appear annually, 95% of which occurs in developing countries, and almost 2 million of the sufferers are believed to die from the disease (2,3). A large number of the infected people bear the latent form of TB which produces a possible risky source of the sickness for the future generations. The HIV epidemic has resulted in the fast increase of the TB pandemic and has enhanced the probability of death from TB. The emergence of multidrug resistant tuberculosis to the firstline drugs such as isoniazid, rifampicin, ethambutol, streptomycin and pyrazinamide has made the disease hard to cure (4–6). This obstacle in the treatment of TB and the statistical facts about its prevalence highlights the necessity of designing novel, more potent and less prone to resistance compounds with fewer side effects.

The design of new anti-tubercular drugs may be accomplished using the sophisticated computational methods which are generally based on two different approaches. Designing new ligands for inhibiting a known biochemical target by noticing its structural features which seems a more ideal approach and proposing novel biologically active compounds after statistical analyses of the structures of some known drugs having the desired biological activities. The first approach is called structure-based drug design, and the other is referred to as ligand-based drug design (LBDD) (7). Quantitative structure activity relationship (QSAR) is a method of the LBDD approach. As one of the most powerful techniques for predicting the bioactivity of various molecules, QSAR starts only with the molecular structure information of the previously reported active molecules. Such a data mining approach offers important insight into the relationships between the molecular structure and biological activity of the investigated compounds by means of statistical models. Various methods have been applied to construct QSAR models including linear and nonlinear regression methods (8,9).

Here we investigate the quantitative structure activity relationships of a series of 49 methyl/trifluoromethoxy 1*H*-indole-2,3-dione-3-thiosemicarbazone derivatives reported in literature as anti-tubercular compounds. Principal component regression (PCR) as a method for feature extraction was employed, and its output was used as the input of linear regression. After reaching the model it was validated using various model validation techniques.

## MATERIALS AND METHODS

### 

#### Data preparation

All calculations were performed in a Pentium IV personal computer (CPU at 2.6 GHz) with Windows XP operating system. All biological data used here were derived from the literature (10). General structures and the structural details of these compounds are reported in Table 1.

**Table 1:** General structures and structural details of 5-methyl/trifluoromethoxy-1*H*-indole-2,3-dione-3-thiosemicarbazone derivatives used in this study. 
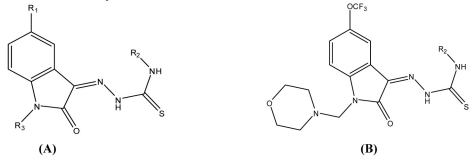

Compound	R_1_	R_2_	R_3_	Type
1	CH_3_	CH_3_CH=CH_2_	H	A
2	CH_3_	C_4_H_9_	H	A
3	CH_3_	C_6_H_5_CH_2_	H	A
4	CH_3_	4-FC_6_H_4_	H	A
5^a^	CH_3_	2-BrC_6_H_4_	H	A
6	CH_3_	3-BrC6H4	H	A
7	CF_3_O	4-NO_2_C_6_H_4_	H	A
8^a^	CF_3_O	CH_3_	H	A
9	CF_3_O	C_2_H_5_	H	A
10	CF_3_O	CH_3_CH=CH_2_	H	A
11	CF_3_O	C_4_H_9_	H	A
12	CF_3_O	*cycl*-C_6_H_11_	H	A
13^a^	CF_3_O	C_6_H_5_CH_2_	H	A
14	CF_3_O	C_6_H_5_	H	A
15^a^	CF_3_O	4-CH_3_C_6_H_4_	H	A
16	CF_3_O	4-CH_3_OC_6_H_4_	H	A
17	CF_3_O	4-FC_6_H_4_	H	A
18	CF_3_O	4-ClC_6_H_4_	H	A
19	CF_3_O	4-BrC_6_H_4_	H	A
20	CH_3_	2-BrC_6_H_4_	CH_3_	A
21	CF_3_O	C_2_H_5_	CH_3_	A
22	CF_3_O	CH_3_CH=CH_2_	CH_3_	A
23	CF_3_O	C_4_H_9_	CH_3_	A
24	CF_3_O	*cycl*-C_6_H_11_	CH_3_	A
25	CF_3_O	C_6_H_5_CH_2_	CH_3_	A
26^a^	CF_3_O	4-CH_3_C6H4	CH_3_	A
27	CF_3_O	4-FC_6_H_4_	CH_3_	A
28	CF_3_O	4-ClC_6_H_4_	CH_3_	A
29	CF_3_O	4-BrC_6_H_4_	CH_3_	A
30	-	CH_3_	-	B
31^a^	-	C_2_H_5_	-	B
32	-	CH_3_CH=CH_2_	-	B
33	-	C_4_H_9_	-	B
34	-	*cycl*-C_6_H_11_	-	B
35^a^	-	C_6_H_5_CH_2_	-	B
36	-	C_6_H_5_	-	B
37^a^	-	4-CH_3_C_6_H_4_	-	B
38	-	4-CH_3_OC_6_H_4_	-	B
39	-	4-FC_6_H_4_	-	B
40	-	4-ClC_6_H_4_	-	B
41	-	4-BrC_6_H_4_	-	B
42	-	4-NO_2_C_6_H_4_	-	B
43	CH_3_	C2H5	H	A
44	CH_3_	*cycl*-C_6_H_11_	H	A
45^a^	CH_3_	C_6_H_5_	H	A
46	CH_3_	4-CH_3_C_6_H_4_	H	A
47	CH_3_	4-ClC_6_H_4_	H	A
48	CH_3_	4-BrC_6_H_4_	H	A
49	CH_3_	C_6_H_5_	CH_3_	A

a Molecules assigned by Kennard and Stone algorithm as test set

pIC_50_ (log 1/IC_50_) is the dependent variable that characterizes the biological parameter for the developed QSAR model. The structures of molecules were drawn and optimized using Hyperchem 7.0 software (11). Semi-empirical AM1 method with Polak-Ribiere algorithm until the root mean square gradient of 0.01 kcal/mol was the optimization method. The resulted geometries were transferred into Dragon program, version 2.1 (developed by Milano Chemometrics and QSAR Group) to calculate some descriptors (12). A large number of theoretical different descriptors were calculated for each molecule. The name and number of calculated descriptors can be seen in the Table 2. These four classes of descriptors were selected to ease the calculations. All the required model development calculations were performed within the MATLAB (version 7.1, MathWorks, Inc.) environment.

**Table 2:** Some descriptors used in model building.

Descriptor	Molecular Descriptor
**Constitutional**	Molecular weight, no. of atoms, no. of non-H atoms, no. of bonds, no. of heteroatoms, no. of multiple bonds (nBM), no. of aromatic bonds, no. of functional groups (hydroxyl, amine, aldehyde, carbonyl, nitro, nitroso, etc.), no. of rings, no. of circuits, no of H-bond donors, no of H-bond acceptors, no. of Nitrogen atoms (nN), chemical composition, sum of Kier-Hall electrotopological states (Ss), mean atomic polarizability (Mp), number of rotable bonds (RBN), etc.
**Topological**	Molecular size index, molecular connectivity indices (X1A, X4A, X2v, X1Av, X2Av, X3Av, X4Av), information content index (IC), Kier Shape indices, total walk count, path/walk-Randic shape indices (PW3, PW4, Zagreb indices, Schultz indices, Balaban J index (such as MSD) Wiener indices, topological charge indices, Sum of topological distances between F..F (T(F..F)), Ratio of multiple path count to path counts (PCR), Mean information content vertex degree magnitude (IVDM), Eigenvalue sum of Z weighted distance matrix (SEigZ), reciprocal hyperdetour index (Rww), Eigenvalue coefficient sum from adjacency matrix (VEA1), radial centric information index, 2D petijean shape index (PJI2), etc.
**Geometrical**	D petijean shape index (PJI3), Gravitational index, Balaban index, Wiener index, etc
**Functional group**	Number of total tertiary carbons (nCt), Number of H-bond acceptor atoms (nHAcc), number of total hydroxyl groups (nOH), number of unsubstituted aromatic C(nCaH), number of ethers (aromatic) (nRORPh), etc.

In this study, the Kennard and Stone algorithm was employed for assigning the training and test sets (13). This method of data splitting has some advantages: the training set molecules map the measured region of the input variable space completely with respect to the induced metric. The other advantage is that the test molecules all fall inside the measured region.

Principal component analysis (PCA) was employed to compress a pool of calculated descriptors into principal components (PCs) as new variables.

As a matter of fact, PCR is a multiple linear regression method which uses the score matrix as new variables for building of the model.

After the building of the model, its validation is a crucial part of any QSAR procedure. In other words, after the calculation of the regression coefficients (b) by the least square methods, the coefficients are used to predict the activity of external test set. For example, if X_c_ and X_t_ are the matrices of factors for calibration and test sets respectively, the Y_c_ and Y_t_ matrices of activity for calibration and test sets can be obtained using the following equations:

Y_c_ = b X_c_

Y_t_ = b X_t_

Matrix of b can be calculated.

The statistical qualities of the generated QSAR models were evaluated using methods such as leave one out cross validation and parameters like predicted residual sum of squares (PRESS) and the root mean square error (RMSE).

Leave one out cross validation is a wellknown and accepted method applied to discover the reliability of the generated QSAR models. Based on this method, a number of modified data sets (equals to the number of the studied molecules) are generated by removing one of the molecules in each case. For each new data set, a model is generated using the modeling procedure applied in the study. Each model is examined through the evaluation of its power in predicting the bioactivity of deleted molecule. This process is repeated until a total set of predicted bioactivity for all of the investigated molecules is achieved. The predictive ability will be evaluated by the cross validation coefficient (*R^2^_cv_*) calculated using the following equation:

$$R_{cv}^{2}\;=\;1-\frac{\overset{n}{\underset{i=1}{\Sigma }}{\left(y_{\exp,i}\;-\;y_{pred,i}\right)}^{2}}{\overset{n}{\underset{i=1}{\Sigma }}{\left(y_{\exp,i}\;-\;\overset{-}{y}\right)}^{2}}$$

Root mean square error of cross validation (RMSECV) for the developed models is reported, as well

Some criteria for the prediction of the model are suggested by Tropsha. If these criteria are satisfied, it can then be concluded that the model is predictive (14).

These criteria include:

*R*^2^ *LOO*>0.5 *R*^2^>0.6

$$\frac{R^{2}\;-\;R_{0}^{2}}{R^{2}}<0.1\;\;\frac{R^{2}\;-\;R_{0}^{'\;2}}{R^{2}}<0.1$$

0.85<k<1.15 or 0.85<k’<1.15

where, *R^2^* is the correlation coefficient of regression between the predicted and observed activities of compounds in training and test set. *R*^2^_o_ is the correlation coefficients for regressions between predicted versus observed activities through the origin, *R*^’2^_o_ is the correlation coefficient for the regressions between observed versus predicted activities through the origin, and the slopes of the regression lines through the origin are assigned by *k* and *k*’, respectively. Details of the definitions of parameters such as *R*^2^_o_, *R*’^2^_o_, *k* and *k*’ are presented in the literature (14).

In addition, according to Roy and Roy(15), it is necessary to study the differences between the values of *R*^2^_o_ and *R*’^2^_o_. They suggested the following modified *R*^2^ form:

$$R_{m}^{2}\;=\;R^{2}\;\left(1-\left|\sqrt{R^{2}\;-\;R_{0}^{2}}\right|\right)$$

If *R*^2^_m_ value for the given model is >0.5, it indicates good external predictability of the developed model.

## RESULTS

**Fig. 1:**
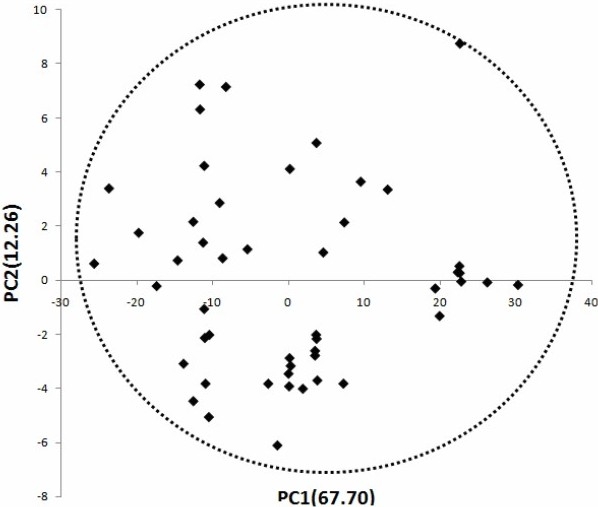
The first two components (PC1, and PC2) from the principal component analysis of the 49 considered molecules.

After deleting zero variance columns of X block, PCA was carried out on the pool of all descriptors. As evident in Table 3, among the generated PCs, only 9 eigenvalue ranked PCs were selected for the next model building. Table 3 demonstrates that PCA gives 9 significant PCs (% variance explained >1) which can explain more than 95.17% of the variances in the original descriptors data matrix. Nine PCs with their eigenvalues are shown in the Table 3. In this Table, the eigenvalues, the percent of variances explained by each eigenvalue, and the cumulative percent of variances are represented. Therefore, the subsequent studies were restricted to these 9 PCs, and the selection of their best subset to perform the linear regression method. Plotting the first PC vs. the second one showed that none of the compounds used in this study were outlier, although 5 clusters existed within the data set (Fig. 1).

**Table 3:** Eigenvslues of calculated PCs, % of explained variances and cumulative variances.

PC No.	Eigenvalue	% variance explained	cumulative variance
1	195.0	60.70	60.70
2	39.46	12.26	72.96
3	27.92	8.672	81.63
4	12.28	3.814	85.45
5	8.755	2.719	88.16
6	8.281	2.572	90.73
7	5.879	1.826	92.56
8	4.508	1.400	93.96
9	3.887	1.207	95.17

When factor scores were used as the predictor parameters in a multiple regression equation using stepwise selection method of PCR, the following equation was obtained:

pIC_50_ = 14.021 (± 2.143) + 12.210 (± 3.216)

f1+ 1.453 (± 0.320) f3

R^2^ = 0.83, S.E. = 0.20, F = 20.05

where, F is F of ANOVA and S.E. is standard error in the resulted model.

The above equation shows high equation statistics (81% explained variance in pIC_50_ data). Since factor scores were used instead of selected descriptors, and each factor-score contains information from different descriptors, loss of information is avoided, and the quality of PCR equation is better than similar equations such as those derived from MLR.

The cross-validation method was used to evaluate the robustness of the proposed model. In leave one out cross validation method, one object at a time was eliminated, and then PCR was performed on the remaining of training set. The activity of the left-out object was predicted using this regression model. This procedure was repeated until each compound in the calibration set was left out once. The optimum number of factors was selected with respect to the quantities of RMSECV, the root mean square error of calibration, and 2 PCs were selected as the optimum number of PCs.

For the evaluation of the predictive power of the generated PCR, the optimized model was applied for the prediction of pIC50 values of all compounds in the calibration and prediction set. The calculated pIC_50_ for each molecule and relative error of prediction by model are summarized in Table 4. Very small values of relative errors confirm the accuracy of the proposed PCR model for modeling the anti-tubercular activity of the studied compounds.

**Table 4:** Calculated activities by PCR and their relative error of prediction (REP).

Compound	Experimental activity	Predicted activity	REP
1	3.743	3.853	0.029
2	5.379	5.124	-0.050
3	3.827	3.554	-0.077
4	4.866	4.983	0.024
5	4.538	4.667	0.028
6	4.941	4.776	-0.035
7	4.882	4.669	-0.046
8	5.115	5.555	0.079
9	5.197	5.544	0.063
10	4.262	4.554	0.064
11	3.478	3.854	0.097
12	4.955	4.459	-0.111
13	3.847	3.454	-0.114
14	4.459	4.836	0.078
15	4.476	4.433	-0.010
16	4.875	4.433	-0.100
17	4.900	4.654	-0.053
18	4.873	4.739	-0.028
19	4.424	4.763	0.071
20	4.260	4.654	0.085
21	4.223	4.530	0.068
22	4.564	4.674	0.024
23	3.390	3.557	0.047
24	3.704	3.640	-0.018
25	4.137	4.718	0.123
26	3.178	3.433	0.074
27	5.063	5.153	0.018
28	4.768	4.454	-0.071
29	5.213	5.460	0.045
30	4.805	4.406	-0.091
31	4.679	4.455	-0.050
32	4.999	4.652	-0.074
33	3.909	3.763	-0.039
34	4.741	4.630	-0.024
35	5.000	4.775	-0.047
36	4.315	4.634	0.069
37	4.862	4.394	-0.107
38	4.619	4.451	-0.038
39	4.633	4.123	-0.124
40	4.608	4.620	0.003
41	4.593	4.535	-0.013
42	4.175	4.285	0.026
43	4.157	4.436	0.063
44	5.152	5.439	0.053
45	4.968	4.529	-0.097
46	4.847	4.453	-0.089
47	4.598	4.440	-0.036
48	3.645	3.439	-0.060
49	3.332	3.237	-0.029

**Fig. 2:**
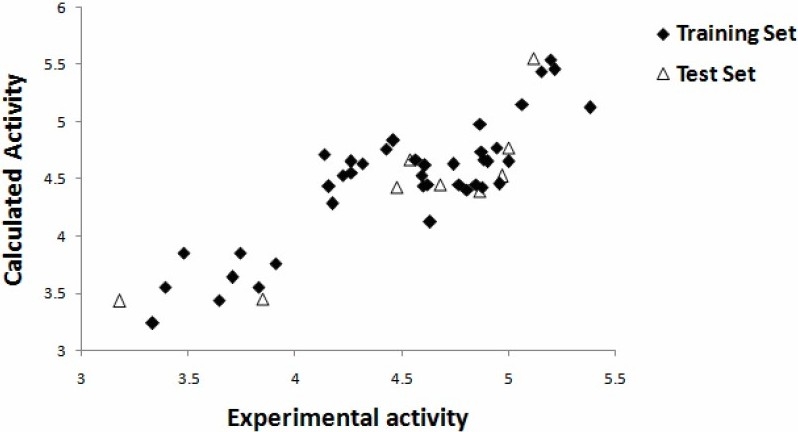
Plot of calculated vs. experimental activity of investigated compounds in training and test sets.

Experimental versus predicted values for pIC_50_ values of training and test sets, obtained by the PCR modeling, are shown graphically in Fig. 2.

Results of various statistical criteria and figures of merit for this model for two subsets of molecules *i.e*. training and test sets are reported in Table 5. The external predictability of a proposed model is generally tested using test sets and *R^2^_CV_*. The satisfactory prediction of the values of the inhibitory activity of test set compounds demonstrates the efficacy of the QSAR in predicting the activities of external molecules. Moreover, the low values of RMSE and PRESS for training, and test sets also add to the statistical significance of the developed models. Besides, on the basis of criteria recommended by Tropsha and also *R_m_^2^* by Roy, the obtained model is very predictive (Table 5).

**Table 5:** Statistical parameters obtained for the developed model for anti-tubrecular activity of the investigated compounds.

Parameter	Training set	Test set
N	40	9
R^2^	0.735	0.762
RMSE	0.284	0.324
PRESS	3.274	0.946
R^2^_LOOCV_	0.792	
RMSE_LOOCV_	0.207	
R^2^_L5O.CV_	0.746	
RMSE_L5OCV_	0.255	
R^2^-R_0_^2^/R^2^	-0.008	-0.041
R^2^-R’_0_^2^/R^2^	-0.009	-0.041
k	1.023	0.987
k’	1.012	0.99
R_m_^2^	0.800	0.711
R^2^ adjusted	0.713	

N: Number of objects in data set, R^2^: Correlation coefficient of experimental and predicted activities, RMSE: Root mean square error:$1/N\left(\sqrt{\Sigma \;{\left(y_{i}\;-\;{ŷ}_{i}\right)}^{2}}\right)$, PRESS: Predicted error sum of square: $\sqrt{\Sigma \;{\left(y_{i}\;-\;{ŷ}_{i}\right)}^{2}}$, R^2^_cv_: Correlation coefficient of leave one out cross validation, RMSEcv: Root mean square error of cross validation

## DISCUSSION

To obtain the effects of the structural features of the studied derivatives on their anti-tubercular activity, QSAR model development was performed with various calculated molecular descriptors (8). Because of the large number of calculated descriptors, PCA was employed to solve the collinearity problem in the generated descriptors, and the PCs were used as new variables for model building. In the PC analysis at first a data pre-processing step must be performed on the descriptors calculated by dragon using autoscaling. Suppose X_i,j_ is the column mean-centered and scaled matrix of descriptors for i samples and j descriptors, and yi,1 the matrix of the activity (pIC_50_). After the generation of principal components, using matrix X_i,j_, the new matrix containing scores of PCs is created. Then these scores are used as new variables for regression. Scores as new variables possess two interesting properties (8): (i) They are sorted as the information content (variance) explaining decreases from the first PC to the last one of the PCs. As a result, the last PCs can be deleted, since they don’t have useful information. (ii) PCs are orthogonal, so the correlation problem that exists in the pool of descriptors calculated in this study is solved.

After the calculation of PCs, these factors are used as new variables in the building of the model. In order to evaluate the final developed model, the existing data set was divided into training and external prediction (test) sets. Almost 20% of the molecules (9 out of 49) were selected as external test set molecules. The training set plays an important role in developing the properties of the model.

The best situation in this stage of the model building is splitting of the data set to guarantee that both training set and test set individually cover the total space occupied by the original data set. The possibility of overfitting of the developed model is increased by the selection of more similar molecules as training set. Hence, ideal splitting of data set can be performed in such a way that each object in the test set is close to at least one of the objects in the training set. One of the best methods for data splitting is using Kennard and Stone algorithm. After dividing the molecules into two parts, training and test sets, based on Kennard and Stone algorithm, building of the regression models using the calibration set was performed.

In order to get the linear relationship with independent variables, logarithms of the inverse of the biological activity (log 1/IC_50_) data of 49 molecules were used.

An exact consideration of different statistical parameters indicated that the developed QSAR model could explain and predict 73% and76% of the variances in the pIC_50_ in training and test sets data, respectively. It was observed that, the plot of data resulted by PCR represents the lowest scattering, with no systematic error As shown in Table 5, *R*^2^ that is an indicative of the goodness of the fitting of the proposed model, was obtained for three sets, and the high value of this parameter indicates a good fitting between the PCs and the predicted values of anti-tubercular activities of the investigated compounds by developed PCR model. This shows the high predictability of the proposed model.

## CONCLUSION

Quantitative relationships between the molecular structure and the inhibitory activity of the series of some 5-methyl/trifluoro-methoxy-1*H* - indole- 2,3- dione- 3-thiosemicarbazone derivatives as anti-tubercular agents were discovered by the collection of the calculated descriptors including topological, geometrical, constitutional, and functional group. As a result, it was found that correctly opted and designed PCR model could practically represent dependence of the 5-methyl/trifluo-romethoxy- 1*H*- indole-2,3- dione- 3-thio-semicarbazone derivatives as anti-tubercular compounds to the extracted PCs from various geometrical, topological, and other calculated descriptors. The optimized principal regression method could simulate the linear relationship between pIC_50_ value and the PCs.
